# Supplementary material for: Direct extraction of lithium from ores by electrochemical leaching
Source: Nat Commun. 2024 Jun 13;15:5066. doi: 10.1038/s41467-024-48867-0 (PMC11176389; doi:10.1038/s41467-024-48867-0)
Supplement: Supplementary file 1 — Supplementary Information [file 41467_2024_48867_MOESM1_ESM.pdf]

Supplementary Information for

Direct extraction of lithium from ores by electrochemical leaching

Hanrui Zhang[1], Ying Han[2], Jianwei Lai[1], Joseph Wolf[1], Zhen Lei[1], Yang Yang[2], Feifei Shi[1, *]

1 John and Willie Leone Family Department of Energy and Mineral Engineering, Pennsylvania State University, University Park, PA 16802, USA.

2 Department of Engineering Science and Mechanics, Pennsylvania State University, University Park, PA 16802, USA.

* Corresponding author

Email address: feifeishi@psu.edu

This supplementary information file includes:

Supplementary Figures 1–26

Supplementary Tables 1–8

Supplementary Notes 1-3

Supplementary References 1-16

Supplementary Movie 1

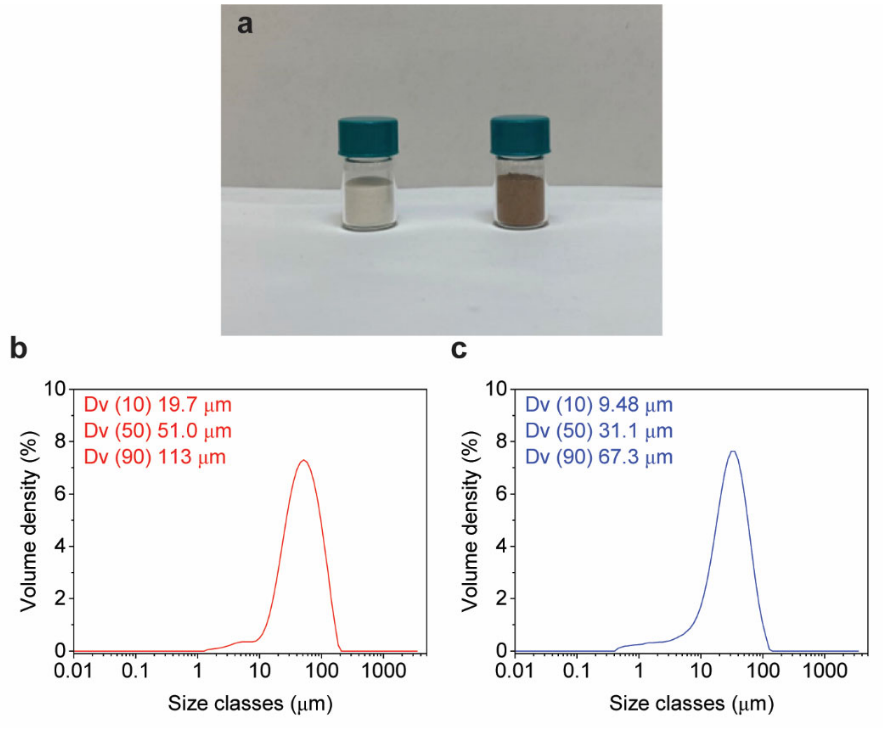

Supplementary Fig. 1. The overview of spodumene particles. **a,** Optical image of α-phase (left) and β-phase (right) spodumene. Size distributions of **b**, α-phase, and **c**, β-phase, measured by laser diffraction.

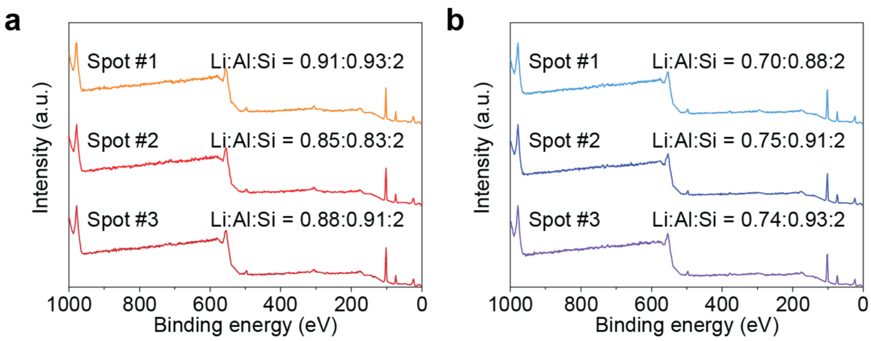

Supplementary Fig. 2. Overall XPS survey spectra. **a,** pristine α-phase, **b**, pristine β-phase. Each sample is repeated 3 times on different spots. Table: Atomic ratio of elements.

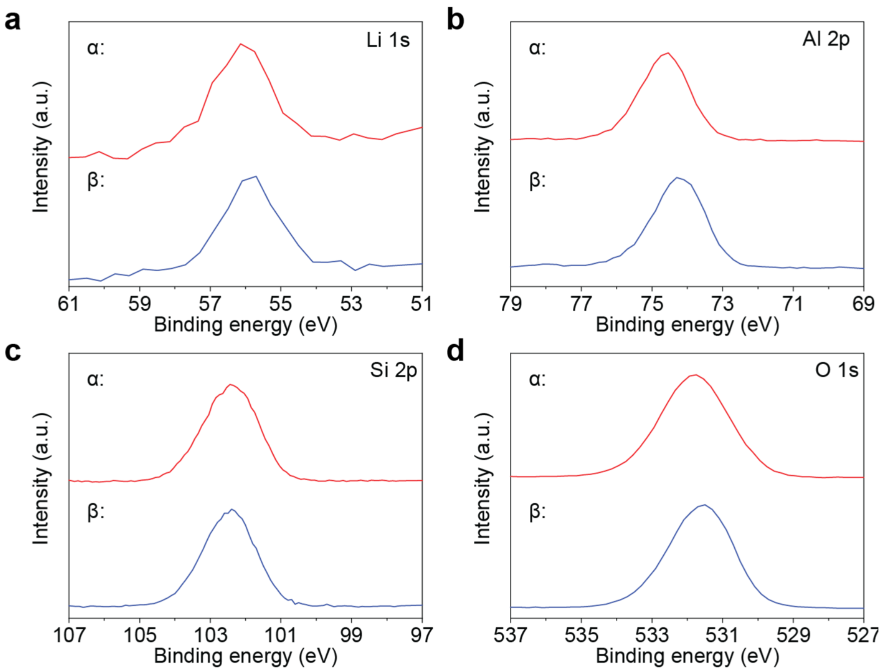

Supplementary Fig. 3. High-resolution XPS spectra centered on **a**, Li 1s, **b**, Al 2p, and **c**, Si 2p and **d**, O 1s on pristine α and β-phase spodumene.

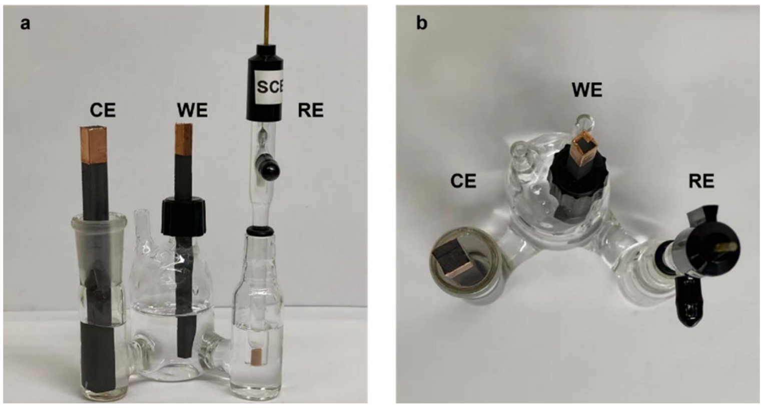

Supplementary Fig. 4. Cell configuration for the small-scale electrochemical test. **a,** side-view and **b,** top-view. The working electrode (WE) is the active material-coated graphite rod, the counter electrode (CE) is a graphite rod (for small-scale leaching) or carbon paper (for scale-up leaching), and the reference electrode (RE) is a saturated calomel electrode (SCE).

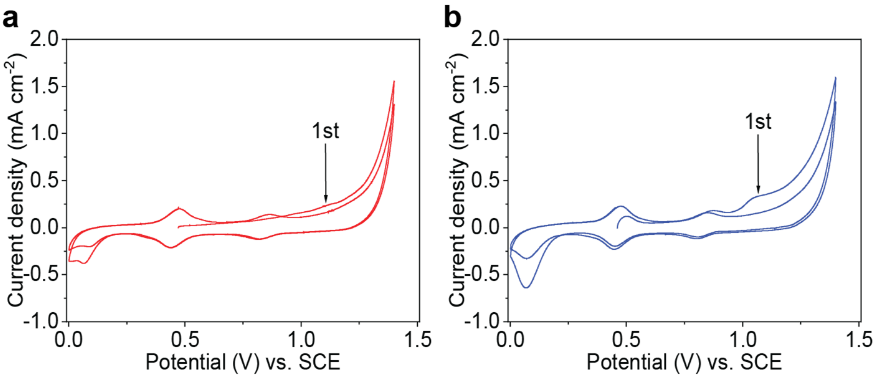

Supplementary Fig. 5. Cyclic Voltammetry (CV) test on spodumene. **a**, α-phase and **b**, β-phase spodumene. Only the first cycle of CV shows the oxidation peak of spodumene. The electrolyte is 0.5 M H_2SO_4 and at a scan rate of 0.5 mV s^{-1}.

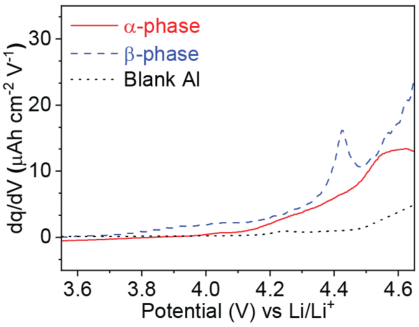

Supplementary Fig. 6. Leaching potential of spodumene versus Li/Li$^+$ in aprotic electrolyte. dq/dV profile of spodumene/Li metal coin cell. The spodumene was coated on an Al current collector with the carbon coating, and a bare current collector was used as a control test. The counter electrode is lithium metal foil and the electrolyte is 1M $LiPF_6$ in ethylene carbonate and diethyl carbonate (EC/DEC 1:1 v/v). The leaching potential of α-phase is 4.59 V vs. Li/Li$^+$, and 4.46 V vs. Li/Li$^+$ for β-phase. The leaching potential difference in the aprotic system (0.13 V) is similar to that in the acidic aqueous electrolyte, indicating the energy barrier of electrochemical leaching does not rely on the concentration of H^+.

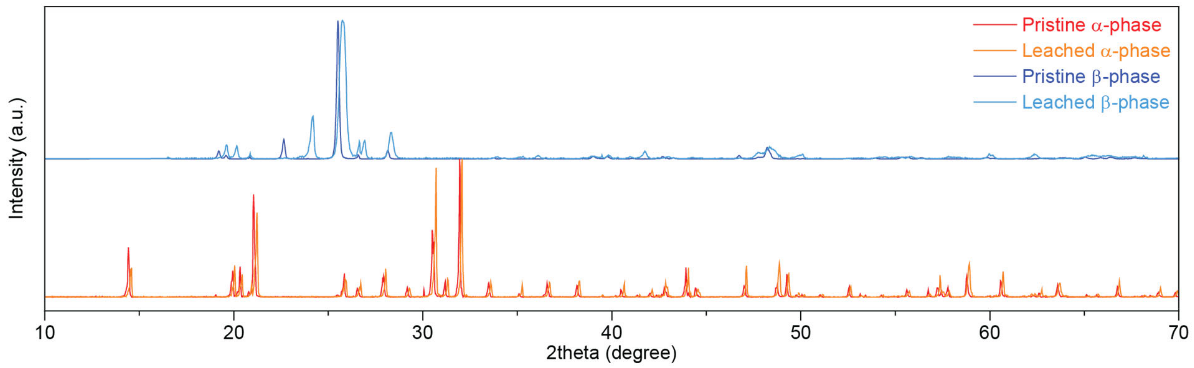

Supplementary Fig. 7. XRD patterns of pristine spodumene samples and spodumene samples after electrochemical leaching at 0.95 V vs. SCE for 24 hours in 0.5 M H_2SO_4 with 0.5 wt. % H_2O_2.

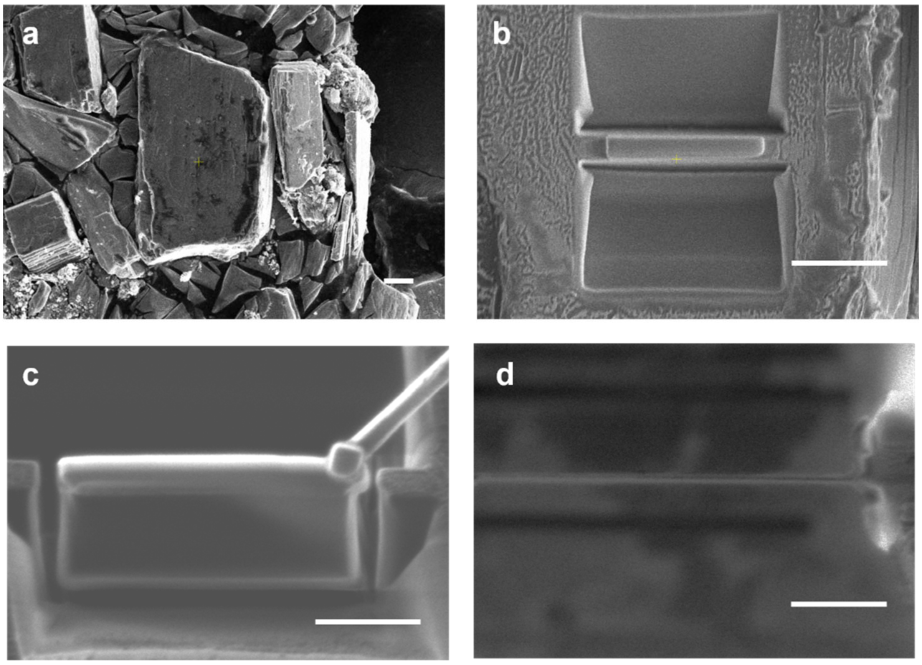

Supplementary Fig. 8. Preparation for TEM imaging sample using FIB lift-out. **a**, SEM image of the selected α-phase spodumene particle. **b**, Trenching and **c**, freeing of the lamella. **d**, the top view of the thinned lamella. Scale bar: **a** – 20 μm, **b** - 10 μm, **c** – 5 μm, **d** – 3 μm.

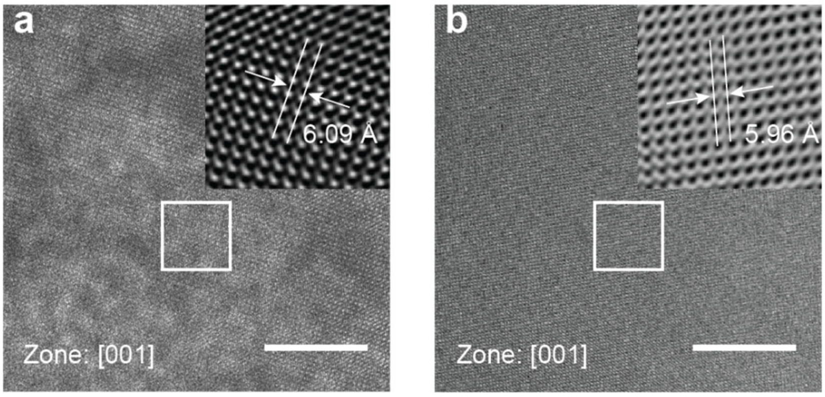

Supplementary Fig. 9. High-resolution TEM (HRTEM) image of α-phase spodumene. **a**, pristine α-phase spodumene and **b**, electrochemically leached α-phase spodumene at 0.95 V vs. SCE for 12 hours. Inset: zoomed-in view of the square area with image filtering to reduce the noise. Scale bars: 20 nm.

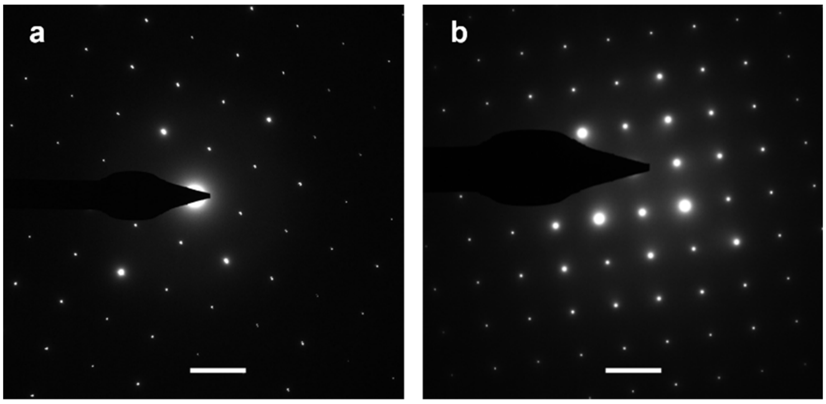

Supplementary Fig. 10. Selected area electron diffraction (SAED) pattern of spodumene. **a**, pristine α-phase and **b**, leached α-phase. Scale bars: 2 nm^{-1}.

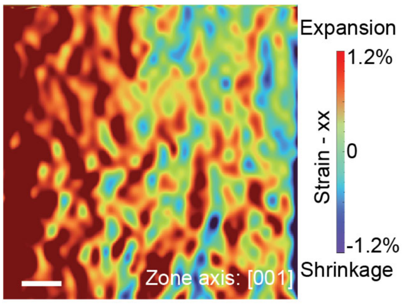

Supplementary Fig. 11. Geometric phase analysis (GPA). GPA analysis of Fig. 2e. This half-leached α-spodumene particle shows the pristine area and leached area with lattice shrinkage of ~ 2%. This shrinkage value is consistent with the calculation from XRD results. The data is processed with Strain++ software.

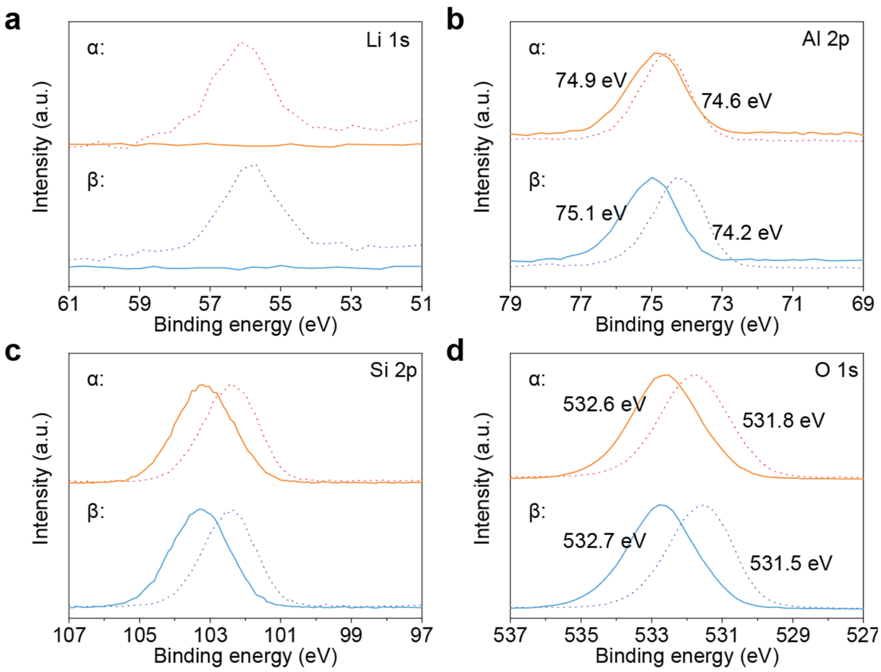

Supplementary Fig. 12. High-resolution XPS spectra centered on **a**, Li 1s, **b**, Al 2p, and **c**, Si 2p and **d**, O 1s of leached α and β-phase spodumene. Dotted lines: pristine α and β-phase. Solid lines: leached α and β-phase.

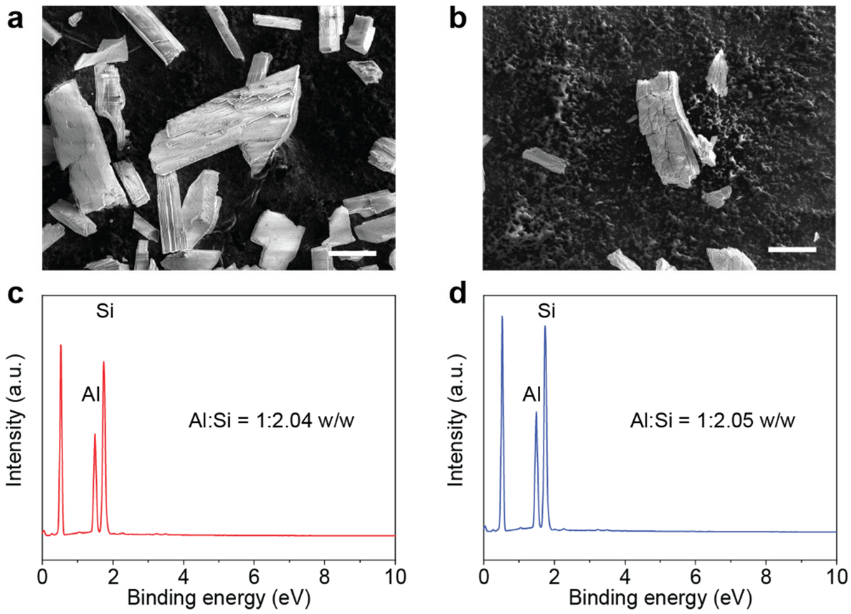

Supplementary Fig. 13. Morphology and element analysis of spodumene after electrochemical leaching. SEM images of electrochemically leached **a,** α-phase spodumene and **b**, β-phase spodumene. EDS spectrum and the elemental ratio of electrochemically leached **c**, α-phase spodumene, and **d**, β-phase spodumene. Scale bar: 10 μm.

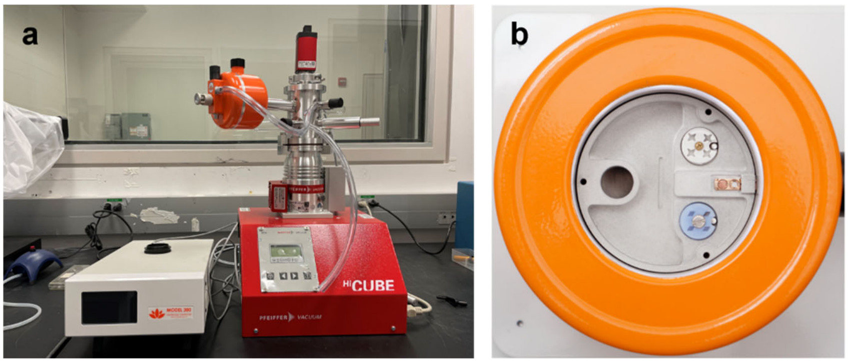

Supplementary Fig. 14. The optical image of **a**, Simple Origin cryo-stage and **b**, Simple Origin loading stage (retrieved from simpleorigin.us).

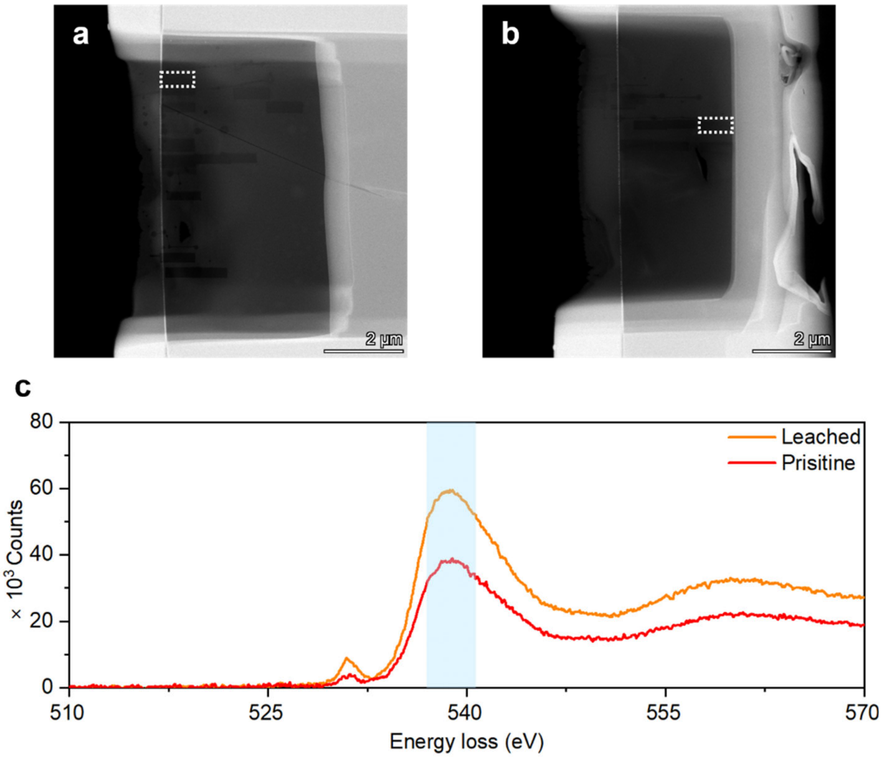

Supplementary Fig. 15. STEM-EELS on pristine/leached α-phase spodumene. TEM image of **a**, pristine α-phase and **b**, leached α-phase. The EELS region is highlighted **c**, EELS spectra of pristine/leached α-phase spodumene, EELS core-loss spectra were normalized against their corresponding zero-loss peak maximum values.

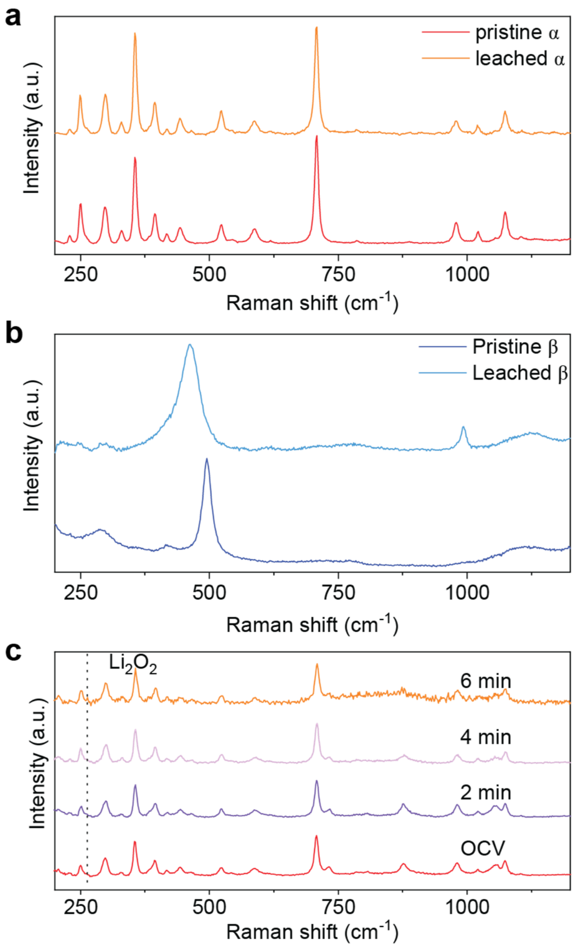

Supplementary Fig. 16. Raman spectra of pristine/electrochemical leached spodumene. **a,** α-phase. **b**, β-phase. **c**, In-situ Raman spectra of α-phase spodumene after electrochemical leaching, voltage is held at 0.8 V vs. SCE during the process. After the electrochemical leaching for ~6 mins, the signal of Li_2O_2 (258 cm^{-1}) starts to show.

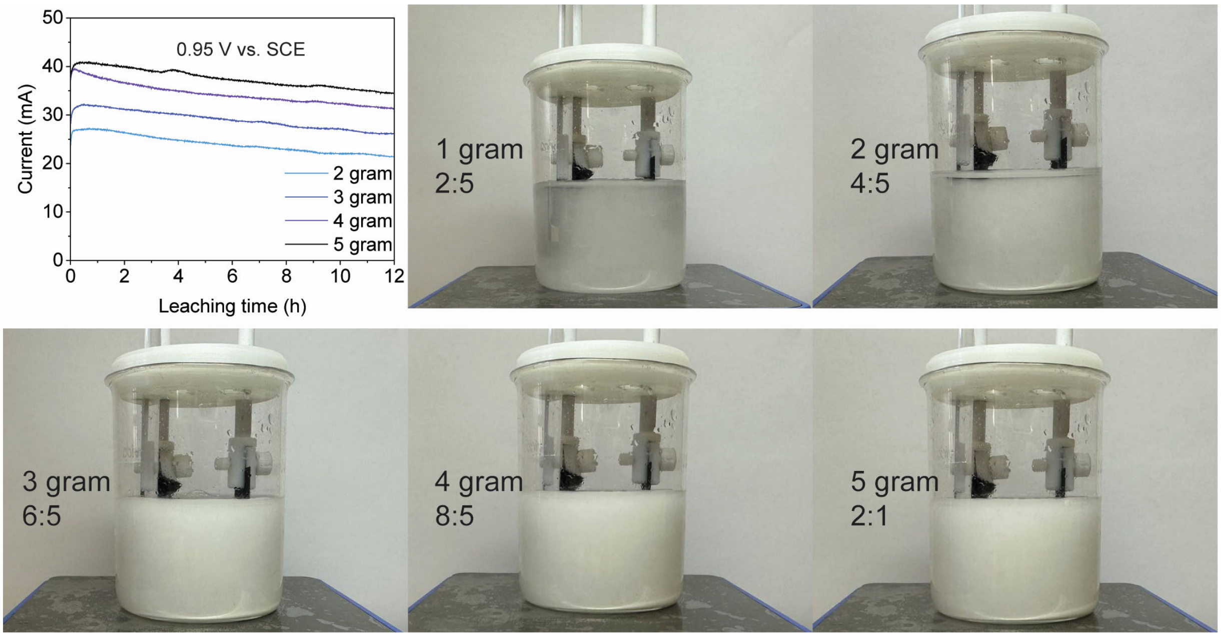

Supplementary Fig. 17. The electrochemical leaching current and optical photos for spodumene: sulfuric acid weight ratio from 2:5 (1g) to 2:1 (5g). The leaching potential is 0.95 V vs. SCE, with the electrolyte as 0.5 M H_2SO_4 with 0.5 wt.% H_2O_2. With the more than loading 3g: 50 ml 0.5 M H_2SO_4 electrolyte, the ratio between acid and active materials is similar to that of the traditional acid leaching procedure (1:1 w/w). This indicates the scale-up potential of electrochemical leaching.

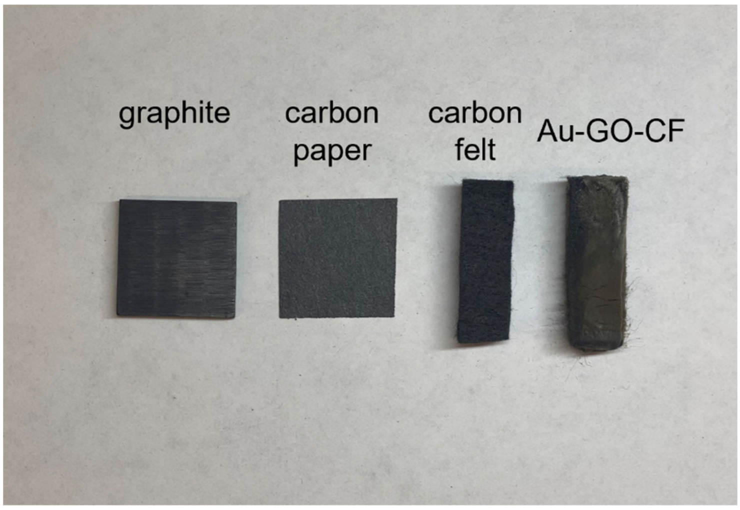

Supplementary Fig. 18. Carbon-based current collectors for electrochemical leaching. From left to right: graphite, carbon paper, carbon felt, and Au-GO-CF current collector.

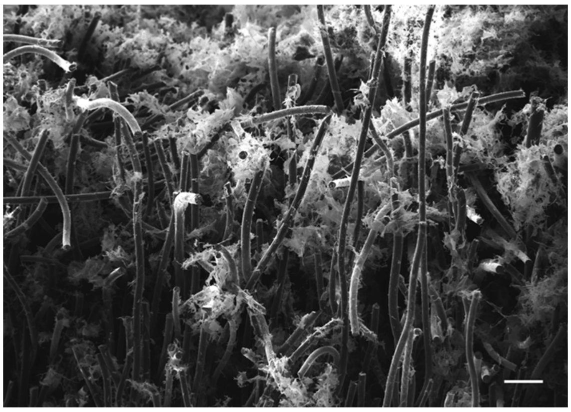

Supplementary Fig. 19. SEM image of Au-GO-CF current collector. Scale bar: 100 μm.

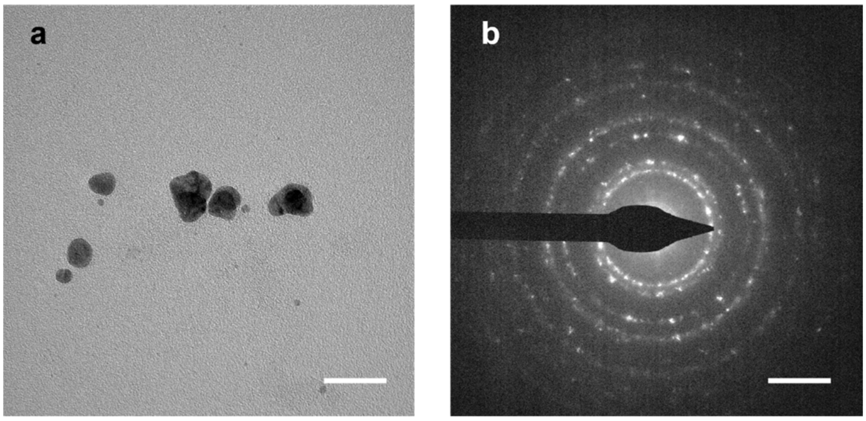

Supplementary Fig. 20. TEM morphology and selective area electron diffraction pattern of gold nanoparticles. **a**, the HRTEM image of Au catalysts on Au-GO-CF, and **b**, selective area electron diffraction (SEAD) of Au catalysts. Scale bars: **a** - 20 nm, **b** - 5 nm^{-1}.

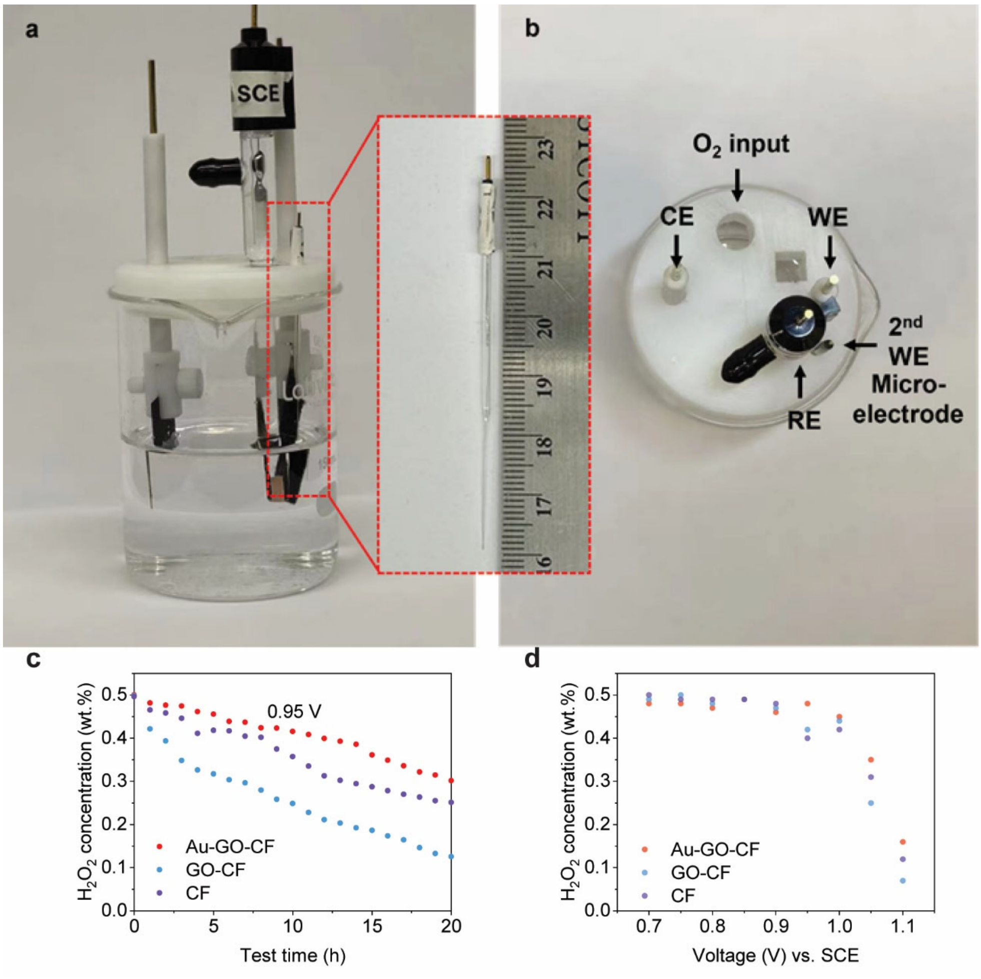

Supplementary Fig. 21. Cell configuration for the scale-up electrochemical test and in-situ monitoring H_2O_2 with microelectrode. **a**, side-view **b**, top-view. The working electrode (WE) is GO-modified porous carbon felt, the counter electrode (CE) is carbon paper, and the reference electrode (RE) is saturated calomel electrode (SCE). A microelectrode is used as the second working electrode for in-situ monitoring of H_2O_2 concentration. De-ionized water is used for a transparent view of the cell setup. The real electrolyte is 0.5 M H_2SO_4 with 0.5 wt.% H_2O_2 and dispersed spodumene particles (opaque color). **c**, localized H_2O_2 concentration as a function of electrochemical leaching time by holding voltage at 0.95 V vs. SCE, corresponding to the concentration in Fig. 3f. **d**, localized H_2O_2 concentration as a function of electrochemical leaching voltage, corresponding to the H_2O_2 concentration in Fig. 3h.

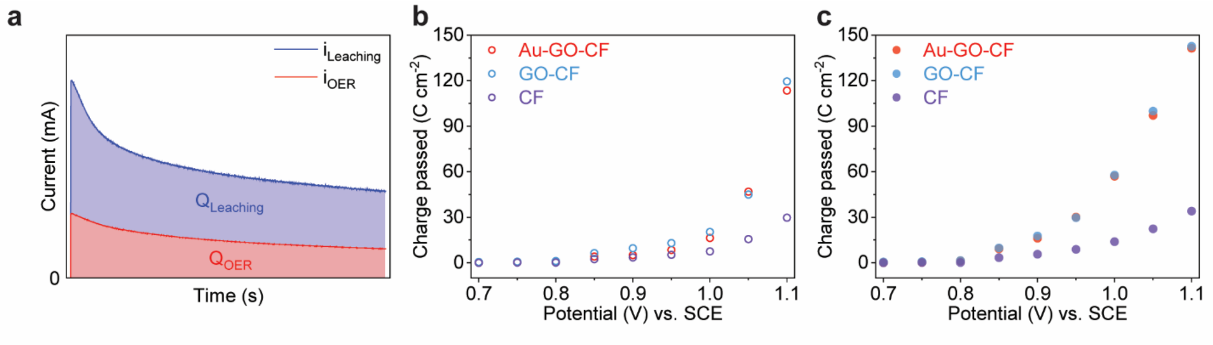

Supplementary Fig. 22. The calculation of Faradaic efficiency. **a**, the schematic shows the charge for leaching (blue) and oxygen evolution reaction(OER) (red). **b**, the hollow circles denote the total charge passed without active materials (Q_{OER}) under various leaching potentials. Each potential was held for 30 mins (without spodumene). **c,** the filled circles denote the total charge passed with spodumene ($Q_{leaching}$ + Q_{OER}) under various leaching potentials. Each potential was held for 30 mins (with 1 g spodumene in 0.5 M H_2SO_4 and 0.5 wt.% H_2O_2). The Faradaic efficiency is calculated by[1]:

$$FE = \frac{Q_{Leaching}}{Q_{Leaching} + Q_{OER}} \quad (1)$$

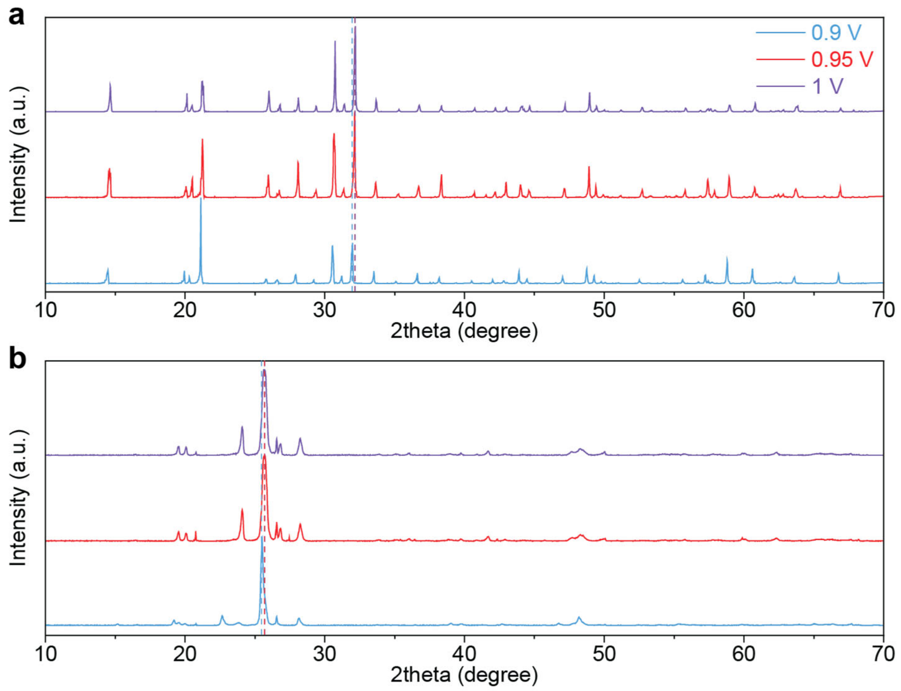

Supplementary Fig. 23. XRD results of leaching residue of spodumene at various leaching voltages. **a**, α- and **b**, β- phase leached at 0.9 V, 0.95 V, and 1 V for 12 hours. The dashed lines denote the (310) of α-phase and (201) of β-phase. The electrolyte is 0.5 M H_2SO_4 with 0.5 wt.% H_2O_2.

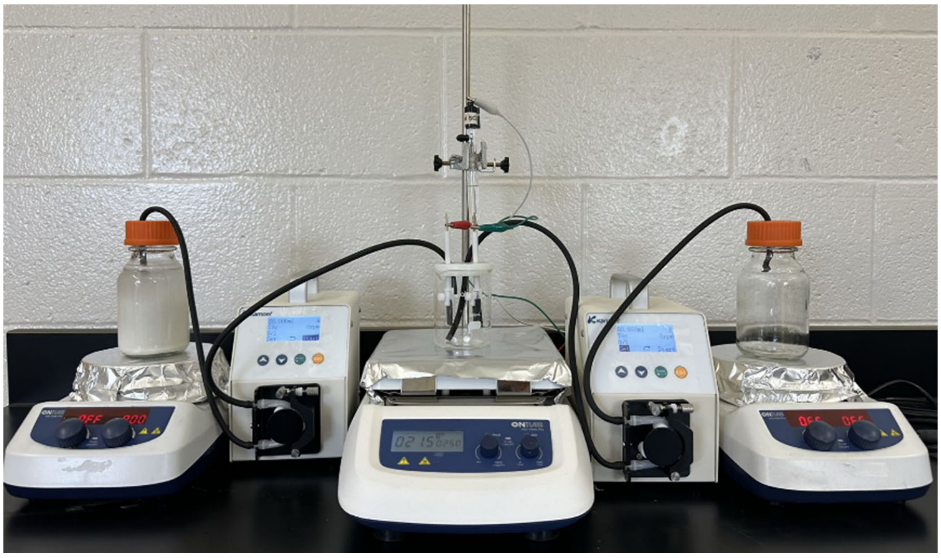

Supplementary Fig. 24. Set-up for flow-mode electrochemical leaching.

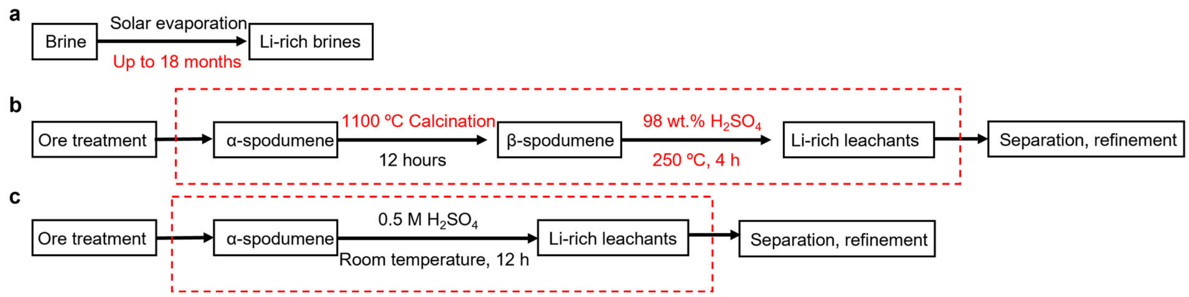

Supplementary Fig. 25. The flow chart of lithium extraction from **a,** brines using solar evaporation, **b**, spodumene using the traditional leaching with sulfuric acid, and **c**, spodumene using the electrochemical leaching (this work).

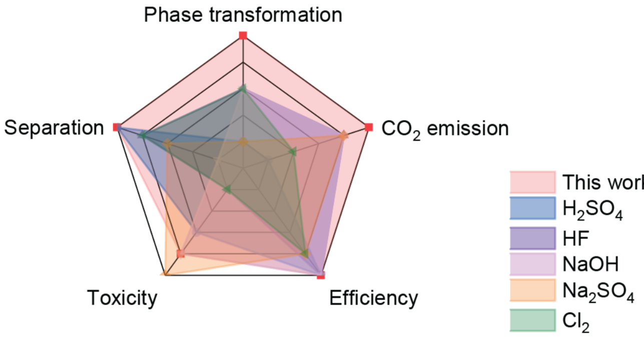

Supplementary Fig. 26. Comparision of the state-of-the-art lithium leaching method. Red-electrochemical method. Blue- industrial sulfuric acid leaching method[2]. Purple-hydrofluoric acid-assisted leaching method[3]. Pink-sodium hydroxide-assisted leaching method[4]. Orange-carbonate/sulfate ion exchange method[5]. Green-chlorination extraction method[6].

Supplementary Table 1. The lithium content of various minerals[7].

**Minerals**	**Chemical formula**	**Lithium content (wt. %)**
**Spodumene**	$LiAlSi_2O_6$	3.7
**Lepidolite**	$K(Li, Al)_3(Al, Si, Rb)_4O_{10}(F, OH)_2$	1.39-3.6
**Petalite**	$LiAlSi_4O_{10}$	1.6-2.27
**Eucryptite**	$LiAlSiO_4$	2.1
**Amblygonite**	$LiAl(PO_4)(F,OH)$	3.4
**Hectorite**	$Na_{0.3}(Mg, Li)_3Si_4O_{10}(OH)_2$	0.54

Supplementary Table 2. The lithium content of α-phase and β-phase spodumene in this work by ICP-AES.

Sample name	Li_2O (wt. %)	Al_2O_3 (wt. %)	SiO_2 (wt. %)
**α-phase pristine**	7.03	26.3	65.0
**α-phase leached**	0.75	28.1	70.6
**β-phase pristine**	7.20	26.6	66.0
**β-phase leached**	0.02	26.0	66.8

Supplementary Table 3. The lattice constant of spodumene samples in this work that calculated from XRD results.

**Sample name**	**a (Å)**	**b (Å)**	**c (Å)**
**Pristine α-phase**	9.456	8.386	5.216
**Leached α-phase**	9.388	8.331	5.241
**Pristine β-phase**	7.541	7.541	9.156
**Leached β-phase**	7.463	7.464	9.093

Supplementary Table 4. The 2θ diffraction angle of pristine and leached spodumene samples in this work.

Crystal plane	2θ – pristine (º)	d – pristine (Å)	2θ – leached (º)	2θ – leached (Å)
**110**	14.515	6.098	14.631	5.050
**200**	19.980	4.441	20.134	4.407
**020**	21.169	4.194	21.315	4.166
**021**	27.988	3.186	28.143	3.169
**220**	29.269	3.049	29.470	3.027
**-221**	30.625	2.917	30.821	2.898
**310**	32.037	2.792	32.265	2.771
**130**	33.579	2.667	33.811	2.649
**-131**	36.632	2.451	36.859	2.436
**221**	38.261	2.351	38.488	2.337

Supplementary Table 5. Chemical composition of the leachant derived from electrochemical leaching and their efficiencies. Each condition is repeated 3 times.

**Leaching potential (V vs. SCE)**	**Feedstock (g)**	**Theoretical concentration (ppm)**	**Concentration from ICP-AES (ppm)**	**Electrochemical leaching efficiency (%)**
**No voltage (24 h)**	1.0	671	0.35	0.05
**0.9**	1.0	671	322	48.08
	1.0	671	310	46.20
	1.0	671	314	46.79
**0.95**	1.0	671	618	92.12
	1.0	671	614	91.51
	1.0	671	607	90.46
**1.0**	1.0	671	604	91.22
	1.0	671	609	90.76
	1.0	671	610	90.90

Supplementary Table 6 Comparison of energy, materials, and cost to produce Li_2CO_3 from traditional chemical leaching and electrochemical leaching.

Resource		Unit price	Chemical leaching[8]				Electrochemical leaching
			Calcination	Roasting	Leaching	Total	
Energy	Electricity (kWh)	0.08[a]	0.19	0.19		0.38	1.58
	Natural gas (m^3)	0.13[b]	0.94	0.30		1.24	
Material (kg)	Water				26.30	26.30	55.92
	Spodumene		8.67			8.67	8.67
	H_2SO_4	0.90[c]		3.02		3.02	2.74
	NaOH	0.32[c]			2.26	2.26	
	Na_2CO_3	0.50[c]			0.55	0.55	
	Kieselguhr	1.00[c]			0.06	0.06	
	HCl	0.15[c]			0.18	0.18	
	H_2O_2	2.11[c]			0.12	0.12	0.07
Cost ($)			0.13	2.77	1.34	**4.24**	**2.73**

a- The price of electricity is $0.0771/kWh (May 2023). Source: U.S. Energy Information Administration (EIA) State Energy Profile Data. https://www.eia.gov/state/data.php?sid=PA

b- The price of natural gas is $3.64/thousand cubic feet for industrial usage (June 2023). Source: U.S. Energy Information Administration (EIA) Natural Gas Prices. https://www.eia.gov/dnav/ng/ng_pri_sum_dcu_nus_m.htm

c- The prices of chemicals were retrieved from Echemi https://www.echemi.com/ Accessed on DATE. The prices listed are for bulk quantities and may vary with region/time.

The spodumene: sulfuric acid ratio is 1:1. By increasing the solid-liquid ratio, the usage of water can be further lowered. The sulfuric acid solution can be recycled for electrochemical leaching.

Supplementary Table 7 Comparison of CO_2 emission to produce Li_2CO_3 from traditional chemical leaching and electrochemical leaching.

Gas Emission	Electricity Source	Chemical leaching[8]			Electrochemical leaching
		Calcination	Roasting	Total	
CO_2 (kg)	Fossil fuel-based power plants [a]	1.90	0.66	2.56	0.632
	Renewable [b]	1.84	0.60	2.44	0
H_2SO_4 (g)[c]			0.05	0.05	0

a- Electricity is generated from the current portfolio of coal and natural gas plants in the U.S. and Average emission rates of CO_2 are calculated by dividing total emission by total electricity generated from coal and natural gas plants in 2021. The emission data for electricity was retrieved from the EIA emission data for electric plants in the United States (2021). Emissions by plant and by region. https://www.eia.gov/electricity/data/emissions/.

b- Here we assume that the electricity usage is from renewables such as wind, solar, etc., without emissions of CO_2.

c- During the traditional chemical roasting process (usually at 250 ºC), gaseous sulfuric acid (H_2SO_4) could be emitted[8].

Supplementary Table 8. State-of-the-art for various leaching methods of Li extraction.

	Phase transformation	Gas emission	Efficiency	Toxicity	Separation
Electrochemical leaching (this work)	No	CO_2	92.2 %	Dilute H_2SO_4	No impurity ions
Sulfuric acid leaching[2]	Mandatory	CO_2, NO_x, SO_2	98 %	Concentrated H_2SO_4	No impurity ions
Hydrofluoric acid leaching[3]	Optional	CO_2, HF	86.8 %	HF	Si^{2+}, Al^{3+}
Sodium hydroxide leaching[4]	Optional	CO_2	95.8 %	NaOH Na_2CO_3	SiO_3^{2-}, AlO_2^-, Na^+
Carbonate/sulfate ion exchange[5]	No	CO_2	93.3 %	Na_2CO_3 Na_2SO_4, NaOH, CaO	Na^+
Chlorination[6]	Optional	CO_2, Cl_2, HCl	89.3 %	Cl_2 gas	Si^{2+}, Al^{3+}

Supplementary Note 1. Monitor the near-current collector concentration of H_2O_2 by a microelectrode.

There are several methods for measuring H_2O_2 concentration, such as titration[9], refractory meter, or commercial H_2O_2 strips[10]. However, these methods only provide the concentration of static bulk solution. By holding the potential below the H_2O_2 reduction potential (-0.65 V vs. SCE), the localized H_2O_2 is reduced by the microelectrode, and the current density is proportional to the H_2O_2 concentrations (Fig. 3g), as reported previously[11,12]. The CV curve for microelectrodes results in a diffusion current plateau (similar to the limiting current in rotating disk electrodes) due to a steady-state response from the 3-D diffusion[13].

Supplementary Note 2. Techno-economic assessment for Li_2CO_3 production from different leaching methods

For both traditional chemical and electrochemical leaching, the pre-treatment of the raw ores (such as the refining) and post-treatment (such as the precipitation) are the same. For the middle stage chemical leaching includes three steps (calcination & roasting & water leaching), while electrochemical leaching is only one step. The chemical usage, energy input, and gas emissions are well documented in the previous literature for the traditional chemical leaching method[8]. Industrial electricity and natural gas prices were retrieved from the U.S. Energy Information Administration (EIA). The prices of the chemicals were retrieved from Echemi[14]. Labor, buildings, and infrastructure are not considered in this study.

Electricity used in electrochemical leaching has two sectors: electricity consumed from leaching voltage and dispersing the spodumene particles. The total charge of electrochemical leaching can be derived from Fig. 3f, which is 179.47 mAh for 12 hours. Based on Fig. 3i, 1g spodumene was fully leached after 12 hours. Hence, the energy consumption from the leaching voltage hold is 0.24 Wh for every gram of spodumene. The voltage between the working electrode and the counter electrode is measured as 1.35 V (when 0.95 V is applied between the working electrode and the SCE reference electrode). Assume electricity for dispersing the spodumene particles is 20% of the electricity consumption for leaching voltage holding. Total electricity usage is around 0.29 Wh /g. To produce 1 kg Li_2CO_3, 1.58 kWh of electricity is needed.

Compared with the traditional chemical leaching method, the as-developed electrochemical leaching method lowers the cost by 35.6%, with significantly less emissions.

Supplementary Note 3. Assessment of various leaching methods for Li extraction.

In the radar graph (Fig. S26), the different indicators are discussed as follows:

CO_2 emission and toxicity[15,16]: During the extraction, some methods need heating assistance to improve the leaching efficiency. Heating will increase the energy input and release gases such as CO_2, SO_2, NO_x, HF, and Cl_2. The electrochemical leaching method doesn't emit any gases other than CO_2 from generating electricity. The sulfate/carbonate ion exchange method and sodium hydroxide leaching emit CO_2 gas from heating. Other methods release toxic gases such as SO_2, HF, and Cl_2.

Toxicity: Some chemical agents are strongly corrosive during the leaching, creating unsafe conditions for workers. The sulfate/carbonate ion exchange method uses unharmful reagents such as Na_2CO_3 salts. Electrochemical leaching and sodium hydroxide leaching use dilute chemicals, which are also safe for workers. Traditional sulfuric acid leaching applies concentrated acid, raising safety concerns. Highly corrosive, toxic reagents (e.g., HF, Cl_2) are used for the hydrofluoric acid leaching and chlorination method.

Separation: After lithium has been leached out, it needs to be separated from other metal ions. An appropriate extraction method should prevent the introduction of additional ions. There is no additional impurity in the leaching process for electrochemical leaching and traditional sulfuric acid leaching. For the hydrofluoric acid leaching and chlorination method, Si^{4+} and Al^{3+} are involved. The sulfate/carbonate ion exchange method will introduce hard-to-sieve Na^+ and K^+ ions. The sodium hydroxide leaching will involve all SiO_3^{2-}, AlO_2^-, and Na^+, as well as other impurities.

Reference

1 Marcandalli, G., Goyal, A. & Koper, M. T. M. Electrolyte Effects on the Faradaic Efficiency of CO(2) Reduction to CO on a Gold Electrode. *ACS Catal* **11**, 4936-4945 (2021). https://doi.org:10.1021/acscatal.1c00272

2 Rioyo, J., Tuset, S. & Grau, R. Lithium Extraction from Spodumene by the Traditional Sulfuric Acid Process: A Review. *Mineral Processing and Extractive Metallurgy Review*, 1-10 (2020). https://doi.org:10.1080/08827508.2020.1798234

3 Rosales, G., Ruiz, M. & Rodriguez, M. Study of the Extraction Kinetics of Lithium by Leaching β-Spodumene with Hydrofluoric Acid. *Minerals* **6** (2016). https://doi.org:10.3390/min6040098

4 Xing, P. *et al.* Lithium Extraction and Hydroxysodalite Zeolite Synthesis by Hydrothermal Conversion of α-Spodumene. *ACS Sustainable Chemistry & Engineering* **7**, 9498-9505 (2019). https://doi.org:10.1021/acssuschemeng.9b00923

5 Kuang, G. *et al.* Extraction of lithium from β-spodumene using sodium sulfate solution. *Hydrometallurgy* **177**, 49-56 (2018). https://doi.org:10.1016/j.hydromet.2018.02.015

6 Fosu, A. Y., Kanari, N., Bartier, D., Vaughan, J. & Chagnes, A. Novel extraction route of lithium from alpha-spodumene by dry chlorination. *RSC Adv* **12**, 21468-21481 (2022). https://doi.org:10.1039/d2ra03233c

7 Meng, F., McNeice, J., Zadeh, S. S. & Ghahreman, A. Review of Lithium Production and Recovery from Minerals, Brines, and Lithium-Ion Batteries. *Mineral Processing and Extractive Metallurgy Review* **42**, 123-141 (2019). https://doi.org:10.1080/08827508.2019.1668387

8 Jiang, S. *et al.* Environmental impacts of lithium production showing the importance of primary data of upstream process in life-cycle assessment. *J Environ Manage* **262**, 110253 (2020). https://doi.org:10.1016/j.jenvman.2020.110253

9 Shi, X., Back, S., Gill, T. M., Siahrostami, S. & Zheng, X. Electrochemical Synthesis of H2O2 by Two-Electron Water Oxidation Reaction. *Chem* **7**, 38-63 (2021). https://doi.org:10.1016/j.chempr.2020.09.013

10 Lu, Z. *et al.* High-efficiency oxygen reduction to hydrogen peroxide catalysed by oxidized carbon materials. *Nature Catalysis* **1**, 156-162 (2018). https://doi.org:10.1038/s41929-017-0017-x

11 Haritha, V. S., Vijayan, A., Sarath Kumar, S. R. & Rakhi, R. B. Voltammetric determination of hydrogen peroxide using MoS2 modified glassy carbon electrodes. *Materials Letters* **301** (2021). https://doi.org:10.1016/j.matlet.2021.130258

12 Guo, Z. *et al.* Facile Strategy for Electrochemical Analysis of Hydrogen Peroxide Based on Multifunctional Fe3O4@Ag Nanocomposites. *ACS Appl Bio Mater* **1**, 367-373 (2018). https://doi.org:10.1021/acsabm.8b00101

13 Sandford, C. *et al.* A synthetic chemist's guide to electroanalytical tools for studying reaction mechanisms. *Chem Sci* **10**, 6404-6422 (2019). https://doi.org:10.1039/c9sc01545k

14 Carretero, D. S., Huang, C. P., Tzeng, J. H. & Huang, C. P. The recovery of sulfuric acid from spent piranha solution over a dimensionally stable anode (DSA) Ti-RuO(2) electrode. *J Hazard Mater* **406**, 124658 (2021). https://doi.org:10.1016/j.jhazmat.2020.124658

15 Ibarra-Gutiérrez, S., Bouchard, J., Laflamme, M. & Fytas, K. Assessing the potential of quebec lithium industry: Mineral reserves, lithium-ion batteries production and greenhouse

gas emissions. *Resources Policy* **74**, 102371 (2021). https://doi.org:https://doi.org/10.1016/j.resourpol.2021.102371
16 Cragg, W., Pearson, D. & Cooney, J. Ethics, surface mining and the environment. *Resources Policy* **21**, 229-235 (1995). https://doi.org:https://doi.org/10.1016/0301-4207(96)85056-2
