# Supplementary material for: Direct extraction of lithium from ores by electrochemical leaching
Source: Nat Commun. 2024 Jun 13;15:5066. doi: 10.1038/s41467-024-48867-0 (PMC11176389; doi:10.1038/s41467-024-48867-0)
Supplement: Supplementary file 3 — Description of Additional Supplementary Files [file 41467_2024_48867_MOESM3_ESM.pdf]

Description of Additional Supplementary Files

Supplementary Movie 1: Scale-up electrochemical leaching using the flow mode.
